# Health-related quality of life among adult patients with cancer in Uganda – a cross-sectional study

**DOI:** 10.1080/16549716.2024.2325728

**Published:** 2024-04-10

**Authors:** Allen Naamala, Lars E. Eriksson, Jackson Orem, Gorrette K. Nalwadda, Zarina Nahar Kabir, Lena Wettergren

**Affiliations:** a Department of Nursing, School of Health Sciences, College of Health Sciences, Makerere University, Kampala, Uganda; b Department of Medical Oncology, Uganda Cancer Institute, Kampala, Uganda; c Department of Neurobiology, Care Sciences and Society, Karolinska Institutet, Huddinge, Sweden; d School of Health and Psychological Sciences, University of London, London, UK; e Medical Unit Infectious Diseases, Karolinska University Hospital, Huddinge, Sweden; f Department of Public Health and Caring Sciences, Uppsala University, Uppsala, Sweden; g Department of Women’s and Children’s Health, Karolinska Institutet, Stockholm, Sweden

**Keywords:** Cancer care facilities, health status, neoplasms, health-related quality of life, Uganda

## Abstract

**Objectives:**

The study aimed to investigate the prevalence and factors associated with poor health-related quality of life in adults with cancer in Uganda.

**Methods:**

This cross-sectional study surveyed 385 adult patients (95% response rate) with various cancers at a specialised oncology facility in Uganda. Health-related quality of life was measured using the EORTC QLQ-C30 in the Luganda and English languages. Predetermined validated clinical thresholds were applied to the instrument in order to identify patients with poor health-related quality of life, that is, functional impairments or symptoms warranting concern. Multivariable logistic regression was used to identify factors associated with poor health-related quality of life in six subscales: Physical Function, Role Function, Emotional Function, Social Function, Pain and Fatigue.

**Results:**

The mean age of the patients was 48 years. The majority self-reported poor functioning ranging between 61% (Emotional Function) to 79% (Physical Function) and symptoms (Fatigue 63%, Pain 80%) at clinically concerning levels. These patients were more likely to be older, without formal education and not currently working. Being an inpatient at the facility and being diagnosed with cervical cancer or leukaemia was a predictor of poor health-related quality of life.

**Conclusion:**

Improvement of cancer care in East Africa requires a comprehensive and integrated approach that addresses various challenges specific to the region. Such strategies include investment in healthcare infrastructure, for example, clinical guidelines to improve pain management, and patient education and support services.

## Paper context

- **Main findings:** The majority of adult patients attending specialized cancer care in Uganda report impaired physical and emotional functioning, and pain, at levels that merit clinical concern.
- **Added knowledge:** The large proportion of in- and outpatients with poor self-reported health-related quality of life indicates supportive care needs, exceeding corresponding figures from high-income settings.
- **Global health impact for policy and action:** Improvement of cancer care in East Africa requires a comprehensive approach that should include investment in healthcare infrastructure, e.g. clinical guidelines to improve pain management, and patient education and support services.

## Introduction

The global cancer burden continues to rise, with marked morbidity and mortality differences among and between countries and by age, gender and cancer types [1]. The global incidence is expected to rise from 19 million cases in 2020 to more than 28 million by 2040 [2]. In 2020, breast cancer among women was the most common diagnosed cancer (11.7% of the total cases) followed by lung (11.4%) colorectal (10%) and prostate cancer (7.3%) [2]. In sub-Saharan Africa, cancer mortality is expected to increase from half a million deaths annually in 2020 to one million per annum by 2030 [3]. In recent years, Uganda has registered a gradual increase of 25% (age-adjusted) in the overall incidence of cancer [4,5]. About 60,000 Ugandans were diagnosed with cancer in 2018, but only 5% of them could access medical services at the Uganda Cancer Institute, which is the only cancer treatment facility in the country [6,7]. The most common cancers in Uganda are cervical (20.5%), breast (8%) prostate (7%) and Kaposi’s sarcoma (11.3%) [2,8].

Cancer and its treatment impact on patients’ health-related quality of life (HRQoL) and is a growing public health concern in sub-Saharan Africa [9–12]. HRQoL is a multidimensional concept that covers the aspects of quality of life that are related to health and illness, and it typically includes physical, role, emotional and cognitive functioning as well as social wellbeing. Since the late 1970s a number of instruments have been developed to assess HRQoL in different domains and or patients with specific conditions [13]. The measurement of HRQoL can be used to identify patients’ needs and provide evidence to inform interventions and clinical decision-making [10,14]. One such measure is the widely used Core Quality of Life Questionnaire (QLQ-C30), which was developed by the European Organisation for Research and Treatment of Cancer (EORTC) [15]. When used together with predetermined thresholds indicating clinical importance, this questionnaire makes it possible to identify patients exhibiting clinically important symptoms or functional impairments that require a health-care professional’s attention [16,17]. There are other approaches that have not been applied in the present study. One such is mapping HRQoL results to preference-based instruments (e.g. EQ-5D), which has shown to be useful in economic evaluations of health-care interventions in high-income countries [18,19].

In East Africa, there are some studies reporting the HRQoL of people with cancer and its associated factors [10,20–25]. A study conducted in Uganda assessed the HRQoL of adult women with ovarian cancer using an abbreviated version of the World Health Organisation Quality of Life instrument [21]. Another study carried out in Uganda and South Africa compared HRQoL in patients with cancer and HIV [26]. Studies have also been undertaken in Tanzania [22,23], Kenya [24], and Ethiopia [10,20,25,27], using the generic EORTC QLQ-C30 [14]. The majority of existing publications are based on data collected at a single institution in samples dominated by women with breast [25,28], cervical [20,23–25] and ovarian cancers [21]. These studies report low overall HRQoL, especially in terms of financial burden, fatigue and pain, particularly in patients with more advanced cancers [20,25,27–29].

Uganda is a country of approximately 47 million people [1]. Traditional lifestyles have been changing and people are living longer and the cancer burden is expected to increase [30]. Health-care services are free in government facilities; however, given the increasing numbers of adult patients with cancer presenting to the country’s resource-poor health-care system, the delivery of optimal cancer care in Uganda is challenging [11,30]. The HRQoL of adults with cancer in Uganda is not well understood, especially for men. This study aims to fill the evidence gap by investigating the prevalence and factors associated with poor HRQoL in adults with cancer in Uganda.

## Methods

### Design and clinical setting

This cross-sectional study was conducted at the Uganda Cancer Institute, a specialised oncology facility. Uganda Cancer Institute is the national referral centre for cancer located in Kampala, the capital and largest city of Uganda with a population exceeding four million inhabitants. In addition to offering chemotherapy, radiotherapy, rehabilitation and palliative care services for in- and out-patients, the Institute undertakes research and provide services in different areas of cancer care, including screening and training for health-care professionals. The Institute sees more than 200 patients daily and has a bed capacity of about 100, with an annual load of 6000–7000 new cases [7].

### Study population

Adult patients (≥18 years) with different types of cancer who spoke Luganda and/or English and who were considered physically and mentally able to participate in an interview (as determined by the head of unit) were approached regarding possible participation in the study. Of the 482 identified patients, 75 were excluded for the following reasons: being critically ill (*n* = 23), having cognitive difficulties (*n* = 5), undergoing staging investigations (*n* = 15) and having language barriers (*n* = 32). A further 22 declined to participate in the study. The remaining 385 patients consented to participate and subsequently answered the EORTC QLQ-C30 (95% response rate) via face-to-face interviews.

### Data collection procedure

Seven registered nurses, holding a bachelor’s degree, collected data during a four-week period (June – July 2019). Information on participants’ sociodemographic (sex, age, marital and employment status, level of education and religion) and clinical characteristics (clinical setting, cancer diagnosis, disease stage and treatment) was collected using study-specific items and medical records.

### EORTC QLQ-C30

The EORTC QLQ-C30 (version 3.0) is a self-reported instrument developed for patients with cancer regardless of its type [15]. The QLQ‑C30 includes 30 items across the following nine scales: Physical Function (five items), Role Function (two items), Emotional Function (four items), Cognitive Function (two items), Social Function (two items), Global Health Status/QoL (two items), Fatigue (three items), Nausea/Vomiting (two items) and Pain (two items). Additionally, six single items measure dyspnoea, sleep disturbances, loss of appetite, diarrhoea, constipation and the financial impact of the disease. With the exception of the Global Health Status/QoL, all the scales have four response alternatives ranging from one to four (‘not at all’, ‘a little’, ‘quite a bit’ and ‘very much’). The Global Health Status/QoL scale includes two items with responses ranging from one (very poor) to seven (excellent). The raw scores of the QLQ-C30 are linearly transformed into zero to 100 point scales. Higher scores on the scales that measure function and the Global Health Status/QoL indicate better functioning and good health status, respectively, while higher scores on the symptom scales represent more symptoms [15].

For the purposes of this study, we translated the EORTC QLQ-C30 (version 3.0) into Luganda and culturally adapted the instrument in accordance with the procedure developed by the EORTC [31,32]. This was followed by an evaluation of the psychometric properties of the versions in Luganda and English, which are the two most common languages in Uganda [33]. The results provided evidence for the validity and reliability of both versions of the EORTC QLQ-C30 for the assessment of HRQoL of adult Ugandans with cancer. All the scales had acceptable Cronbach’s values, ranging between 0.79 and 0.96 [33], except for the cognitive scale (Luganda α = 0.66, English α = 0.50). This scale was excluded from the present study.

It has been suggested that the EORTC QLQ-C30 transformed scores (0–100) are better interpreted using thresholds to indicate clinical importance [16,17]. Such an approach makes it possible to identify patients exhibiting clinically important symptoms or functional impairments that require a health-care professional’s attention (i.e. clinical case) [16]. To develop these thresholds, Giesinger et al. [17] interviewed patients and healthcare professionals to obtain their views on what makes a symptom or functional impairment clinically important. Three aspects were found to reflect clinical importance namely: being limited in everyday life, having the need for (healthcare) help and having a health problem/symptom that causes the patient or family/partner to worry. Using this as a basis, they developed anchor items for each QLQ-C30 subscale (with domain-specific wording). Clinically relevant cases were identified as those where participants responded ‘quite a bit’ or ‘very much’ (using the same response scale as the QLQ-C30) to at least one of the anchor items in the respective scale [16,17]. The thresholds were established following an analysis of data of patients from European countries with mixed diagnoses and treatments, and high diagnostic accuracy was demonstrated when identifying functional health impairments and clinically important symptoms [17]. The thresholds suggested by Geisinger et al. for the functional and symptom scales vary by scale and range from below 58 (Role and Social Function) to below 83 (Emotional Function). For the symptom scales, the scores indicating a clinically important symptom vary from above 25 (Pain) to above 39 (Fatigue).

### Statistical analyses

Data were analysed using Stata statistical software version 15 [34]. The descriptive data (complete cases) are presented as means, standard deviations, frequencies, and percentages. Student’s *t*-test was used to determine possible differences in the means of the QLQ-C30 scales between men and women. Predefined validated thresholds were applied to identify patients exhibiting clinically important symptoms or functional impairments (clinical cases) [16] defined as poor HRQoL in the current study. In addition to presenting percentages for those rating poor HRQoL across subscales, six logistic regression models were conducted to identify the factors associated with rating poor HRQoL in the selected QLQ-C30 scales of Physical Function, Role Function, Social Function and Emotional Function as well as those of Fatigue and Pain (dependent variables).

The choice of independent variables was informed by the literature [23,28,30]. The following sociodemographic factors were selected as independent variables: age (continuous), sex (male/female), education level (none/primary/secondary/tertiary) and occupation (not working/student/business/employed). The following clinical factors were also included as independent variables: clinical setting (inpatient/outpatient); cancer stage (early stage [I – II]/late-stage [III – IV]); palliation (yes/no); active cancer treatment (yes/no); and cancer diagnosis. Factors that were significant in the bivariate analyses (*p* ≤ 0.05) were further analysed in the multivariable logistic regression. All tests were two tailed with *p* ≤ 0.05 considered significant.

## Results

A high proportion of adult patients in specialised cancer care in Uganda reported poor health-related quality of life with regard to physical (79%) and mental functioning (61%). Four out of five reported pain. Sociodemographic and clinical characteristics of the participants are presented in Table 1. Participants’ ages ranged between 18 and 89 years (mean 47.5 ± 15.7 years). More than 80% of the participants had some form of education and had a source of income through employment or business ownership. Most of the patients were hospitalised (67%), and almost three-quarters (72%) had advanced cancer. Nearly a quarter (24%) had cervical cancer, and almost half (46%) were receiving chemotherapy.

**Table 1.** Sociodemographic and clinical characteristics of patients in specialised cancer care in Uganda (N = 385).

Age in years, mean (SD)	47.5 (15.7)	
Participants’ characteristics	n	%
**Sex**		
Male	131	34.0
Female	254	66.0
**Marital status**		
Married/cohabiting	217	56.4
Divorced/widowed	106	27.5
Never married	62	16.1
**Education level**		
None	54	14.0
Primary	143	37.1
Secondary	123	31.9
Tertiary	65	16.9
**Religion**		
Christian	332	86.2
Muslim	48	12.5
Others	5	1.3
**Occupation**		
Employed	86	22.3
Business owner	237	61.6
Student	14	3.6
Not working	48	12.5
**Clinical setting**		
Inpatient	257	66.8
Outpatient	128	33.2
**Clinical stage**		
Early (I–II)	106	27.5
Late (III–IV)	279	72.5
**Treatment**		
Chemotherapy only	179	46.5
Chemotherapy and radiotherapy	56	14.5
Radiotherapy only	38	9.9
No therapy	112	29.1
Palliation	21	5.5
Surgery	38	9.9
**Cancer diagnosis**		
Cervical cancer	92	23.9
Breast cancer	68	17.7
Kaposi’s sarcoma	43	11.2
Leukaemia	26	6.8
Prostate cancer	22	5.7
Oesophageal cancer	20	5.2
Lymphoma	13	3.4
Other cancers^a^	101	26.2

^a^Other cancers include lung cancer (*n* = 8), ovarian cancer (*n* = 8), colon cancer (*n* = 5), malignant melanoma (*n* = 5), oral cancer (*n* = 4), stomach cancer (*n* = 4), head and neck cancers (*n* = 4) and other cancers (*n* = 63).

The mean values of the EORTC QLQ-C30 scales by sex are presented in Table 2. There were no statistically significant differences in HRQoL between the sexes.

**Table 2.** Mean values of the EORTC QLQ-C30 scales as reported by patients in specialised cancer care in Uganda.

QLQ-C30 scales	All (*N* = 385) Mean (SD)	Men (*n* = 131) Mean (SD)	Women (*n* = 254) Mean (SD)	*t*-values	*p*-values*
Global Health Status	49.7 (25.2)	50.0 (24.2)	49.6 (25.8)	0.145	0.885
Physical Function	53.4 (31.0)	53.6 (29.6)	53.3 (31.8)	0.099	0.921
Role Function	36.8 (35.3)	36.6 (37.7)	36.8 (34.1)	−0.044	0.964
Emotional Function	57.5 (32.3)	61.9 (32.5)	55.2 (32.1)	1.937	0.053
Social Function	31.6 (33.5)	32.7 (35.0)	31.1 (32.8)	0.442	0.659
Nausea/Vomiting	24.4 (33.7)	22.6 (34.2)	25.3 (33.5)	−0.722	0.471
Fatigue	53.0 (32.3)	51.7 (33.4)	53.6 (31.7)	−0.532	0.595
Pain	59.1 (34.5)	57.1 (34.8)	60.2 (34.3)	−0.821	0.412
Dyspnoea	19.8 (33.3)	20.9 (33.2)	19.3 (33.4)	0.439	0.661
Insomnia	39.7 (38.2)	39.4 (38.1)	39.8 (38.4)	−0.079	0.937
Appetite Loss	41.8 (39.2)	38.2 (39.0)	43.7 (39.2)	−1.313	0.190
Diarrhoea	12.8 (27.3)	11.5 (26.1)	13.5 (27.9)	−0.704	0.482
Constipation	23.5 (36.7)	23.7 (38.9)	23.4 (35.6)	0.077	0.939
Financial Difficulties	89.7 (24.8)	90.8 (24.5)	89.1 (25.0)	0.649	0.517

*Student’s *t-*test was used to assess the differences between men and women.

### Prevalence of poor HRQoL (clinical cases)

The proportion of patients who reported HRQoL at levels warranting concern with poor HRQoL (clinical cases) in the functional scales ranged between 61% (Emotional Function) and 79% (Physical Function), and for the symptom scales, the proportion ranged between 22% (Diarrhoea) and 80% (Pain), see Table 3.

**Table 3.** EORTC QLQ-C30 mean scale scores for patients reporting poor HRQoL (clinical cases) and those with acceptable/high ratings (non-cases) in specialised cancer care in Uganda.

QLQ-C30 scales	Poor health-related quality of life (clinical cases)			Non-cases		
	Mean (SD)	n	%	Mean (SD)	n	%
Physical Function	42.4 (24.9)	305	79.2	95.4 (5.7)	80	20.8
Role Function	18.0 (18.5)	280	72.7	86.7 (15.2)	105	27.3
Social Function	16.0 (19.6)	293	76.1	81.3 (15.0)	92	23.9
Emotional Function	36.2 (22.6)	233	60.5	90.1 (9.9)	152	39.5
Nausea/Vomiting	54.6 (29.8)	172	44.7	0 (0)	213	55.3
Fatigue	73.7 (19.8)	242	62.9	17.9 (13.4)	143	62.9
Pain	72.2 (24.6	309	80.3	6.1 (8.1)	76	19.7
Dyspnoea	64.1 (27.2)	119	30.9	0 (0)	266	69.1
Insomnia	82.9 (16.7)	150	39.0	12.1 (16.1)	235	61.0
Appetite Loss	84.7 (16.7)	157	40.8	12.3 (16.1)	228	59.2
Diarrhoea	59.4 (26.1)	83	21.6	0 (0)	302	78.4
Constipation	85.3 (16.6)	91	23.6	4.3 (11.2)	294	76.4
Financial Difficulties	93.8 (15.9)	368	95.6	0 (0)	17	4.4

### Factors associated with poor HRQoL (clinical cases)

The results of the multivariable logistic analysis are presented in Table 4. Higher age was associated with poor HRQoL in the following three scales: Physical Function (AOR: 1.02, 95% CI: 1.01–1.04; *p =* 0.01) and Role Function (AOR: 1.02, 95% CI: 1.01–1.04; *p =* 0.02) as well as Pain (AOR: 1.04, 95% CI: 1.01–1.06; *p* = 0.001). Patients with secondary education were less likely to report poor HRQoL in the Emotional Function (AOR: 0.23, 95% CI: 0.09–0.55; *p* < 0.01) and Fatigue (AOR: 0.49; 95% CI: 0.24–0.99; *p* = 0.05) scales than those with no formal education. Similarly, patients with tertiary education (AOR: 0.27, 95% CI: 0.10–0.72; *p* = 0.01) were less likely to report poor HRQoL in the Emotional Function scale than their counterparts with no formal education. Additionally, patients who did not have any occupation (not working) were more likely to report poor HRQoL in the Social Function scale (AOR: 3.70, 95% CI: 1.02–13.36; *p* = 0.05).

**Table 4.** Factors associated with poor health-related quality of life in adult patients with cancer in Uganda.

		Physical Function	Role Function	Emotional Function	Social Function	Fatigue	Pain
	n	AOR (95% CI)	AOR (95% CI)	AOR (95% CI)	AOR (95% CI)	AOR (95% CI)	AOR (95% CI)
Age (years)	385	1.02 (1.01–1.04)**	1.02 (1.01–1.04)*				1.04 (1.01–1.06)**
Education level							
None (ref)	54						
Primary	143	1.14 (0.47–2.78)	0.87 (0.33–2.31)	0.44 (0.18–1.07)		0.95 (0.48–1.89)	0.78 (0.30–2.04)
Secondary	123	0.78 (0.32–1.88)	0.66 (0.24–1.83)	0.23 (0.09–0.55)**		0.49 (0.24–0.99)*	0.62 (0.22–1.71)
Tertiary	65	0.44 (0.17–1.13)	0.35 (0.12–1.02)	0.27 (0.10–0.72)**		0.51 (0.23–1.15)	0.44 (0.15–1.30)
Occupation							
Employed (ref.)	86						
Business	237				1.84 (0.95–3.57)		
Student	14				3.20 (0.39–26.28)		
Not working	48				3.70 (1.02–13.36)*		
Clinical setting							
Outpatient (ref)	128						
Inpatient	257		2.99 (1.70–5.26)**				
Clinical stage							
Early (I-II) (ref)	106						
Late (III-IV)	279					1.44 (0.89–2.33)	
Palliation							
No (ref)	364						
Yes	21			6.32 (0.97–41.17)		2.85 (0.77–10.56)	
Active cancer treatment							
No (ref)	273						
Yes	112			0.64 (0.37–1.12)			0.71 (0.36–1.39)
Cancer diagnosis							
Breast (ref.)	68						
Cervix	92			3.20 (1.50–6.81)**		1.71 (0.87–3.39)	2.02 (0.85–4.77)
Kaposi’s sarcoma	43			1.64 (0.70–3.87)		0.88 (0.40–1.94)	1.52 (0.62–3.74)
Leukaemia	26			2.70 (0.95–7.65)		3.82 (1.22–11.97)*	2.54 (0.51–12.76)
Prostate	22			1.02 (0.38–2.75)		1.23 (0.45–3.39)	0.30 (0.09–0.96)*
Oesophageal	20			1.83 (0.66–5.12)		1.49 (0.52–4.25)	1.14 (0.38–3.43)
Lymphomas	13			1.20 (0.36–3.96		0.90 (0.25–3.19	1.96 (0.39–9.93)
Other cancers	101			2.28 (1.14–4.57)**		2.27 (1.16–4.44)*	3.86 (1.62–9.20)**

Each model included only the factors that were associated with the specific subscale in the bivariate analysis *p ≤ 0.05, **p < 0.01.

Among the clinical factors, the regression analyses indicated that being an inpatient was significantly associated with poor HRQoL with regard to Role Function (AOR: 2.99, 95% CI: 1.70–5.26; *p* < 0.001). Compared to those with breast cancer, patients with cervical cancer were more likely to report poor HRQoL with regard to Emotional Function (AOR: 3.20, 95% CI: 1.50–6.81; *p <* 0.001). Furthermore, patients diagnosed with leukaemia were almost four times more likely than those with breast cancer to report poor HRQoL in the Fatigue scale (AOR: 3.82, 95% CI: 1.22–11.97; *p* = 0.02). Patients with other types of cancers (*n* = 101) reported poor HRQoL to a greater extent than those with breast cancer in the following three subscales: Emotional Function (AOR: 2.28, 95% CI: 1.14–4.57; *p* = 0.02), fatigue (AOR: 2.27, 95% CI: 1.16–4.44; *p* = 0.02) and Pain (AOR: 3.86; 95% CI: 1.62–9.20; *p* < 0.001). Patients with prostate cancer were less likely to report pain than those with breast cancer (AOR: 0.30; 95% CI: 0.09–0.96); *p* < 0.001).

## Discussion

In this study, a high proportion of the adult patients attending a specialised cancer facility in Uganda reported impaired physical, role, emotional, or social functioning (61–79%) and symptoms of fatigue and pain and (63–80%) at levels of clinical concern. Almost all patients reported financial difficulties (96%). Factors associated with poor HRQoL were older age, no formal education and not currently working. In addition, being an inpatient diagnosed with cervical cancer or leukaemia were associated with poor HRQoL. These results indicate poorer HRQoL than what has been reported by patients with cancer in European studies [17] and some other studies in low-income countries [22,28,35]. This discrepancy could be attributed to the poorly resourced health facilities and the high proportion of patients with advanced cancer in this study sample. In Uganda, approximately 40% of the population lives in extreme poverty [36–39] and a diagnosis of cancer in a family will, apart from a loss of income, lead to additional family costs with regard to transport to hospital, food and medications [3,20,22,40,41].

Our results are consistent with previous studies of HRQoL in adult patients with cancer in East African countries in which the same instrument, namely EORTC QLQ-C30, was used [20,22–24,28]. However, there were some differences. Mean scores on the Emotional Function scale differed (>10 points) between countries. In Tanzania, patients indicated better functioning (mean 71.8) [22] compared with those in this study (mean 57.5), while in Ethiopia, patients reported worse emotional functioning (mean 45.9) [28]. Additionally, patients in Ethiopia had higher levels of fatigue (mean 65.2) compared with patients in this study (mean 53.0). Between study differences in HRQoL may be due to variations in types of patients and levels of illness [23,25]. For example, Mvunta et al. [23] assessed women with cervical cancer after completion of chemoradiotherapy. In that study, 70% were diagnosed at an early stage and those with comorbidities were excluded. Similarly, Sibhat et al. [25] studied Ethiopian women with breast cancer at an outpatient unit. Of those samples, half had received surgical treatment and none had comorbidities.

The fact that four out of five patients in our study reported pain at a level that corresponded with poor HRQoL is of concern. The literature on the management of cancer-related pain provides a deeper understanding of the factors associated with pain in people with cancer [42]. The barriers for optimal pain management in low-income countries include poor pain reporting channels, misunderstandings of pain leading to under-treatment, and administrative, professional, judicial, and economic impediments [42]. They include inadequate education of health-care professionals (insufficient knowledge and a poor understanding of the use of analgesics), limited facilities for pain management, fear of side effects, misconceptions about pain medications (e.g. fear of addiction) and poor access to pain medications [42].

The National Comprehensive Cancer Network (NCCN) Clinical Practice Guidelines in Oncology (NCCN Guidelines®) provide recommendations for the management of cancer-related pain in adults and are intended to assist health professionals in cancer care when monitoring the treatment of pain in these patients [43]. The Guidelines include the following five dimensions: analgesia (‘has there been a reduction in pain?’); activity (‘has the patient improved in functioning?’); adverse effects (‘is the patient experiencing adverse effects?’); aberrant behaviours (‘does the patient show signs of medication misuse?’); and affect (‘does the pain have an impact on the patient’s mood?’). Additionally, they advise all care providers to consider assessing pain using three pain levels: mild (1–3), moderate (4–7) and severe (8–10) [43]. The successful implementation of the NCCN Guidelines requires capacity building, which includes providing training for health-care workers in cancer care. In addition to the development and implementation of policies, there needs to be increasing awareness and knowledge among patients and communities regarding cancer treatment regimens, their side effects and pain management.

We identified some clinical factors that were associated with a poor HRQoL. Patients with cervical cancer, leukaemia and other cancers were more likely to report poor HRQoL (i.e. clinical cases) in the Emotional Function, Fatigue and Pain scales, respectively, while patients with prostate cancer were less likely to report pain compared to those with breast cancer. The group of other cancers included patients diagnosed with lung cancer, ovarian cancer, colon cancer, malignant melanoma, oral cancer and, head and neck cancers. Our findings revealed that poor HRQoL was directly associated with increasing age with regard to Physical and Role Function as well as Pain, while patients with secondary and tertiary education, compared to those with no formal education, reported better HRQoL, which is in line with research from high-income countries [44–46]. A possible explanation for this is that literate and well-educated patients have the capacity and resources to access care [27,47]. Poor health-care-seeking behaviours, advanced stage at presentation, dearth of treatment choices and poor treatment compliance are known to be related to no or low levels of education [28,48]. Additionally, lack of occupation (not working) was significantly associated with poor Social Function. Patients who are not working face socioeconomic challenges because they depend entirely on relatives and friends for income, transport to hospital and buying drugs. Since cancer is a long-term condition, helpers may become tired and finally abandon these patients [30,41,49]. We therefore recommend cancer awareness strategies to inform the public and communities about cancer and its treatment. These will empower them with relevant knowledge to improve social support and reduce stigmatisation among people with cancer in Uganda. There is a need for research to better understand how these patients perceive their social situation. This can then better inform culturally appropriate, patient-centred care for people with cancer in order to meet their preferences and needs regarding care [50].

Sixty-two per cent of the patients in our study reported cancer-related fatigue, which is a symptom commonly experienced by patients during and after active cancer treatment [42,51].

Being fatigued has deleterious effects on life and is not adequately addressed by health-care providers globally [51]. Patients with fatigue are more likely to have greater financial stress, higher utilisation of health-care services and increased risk for mortality. Currently, no gold standard exists for the management of cancer-related fatigue [51,52]. Exercise has been reported to be safe and well tolerated during and after cancer treatment and been shown to improve physical fitness and quality of life [52]. Essentially, patients should be encouraged to avoid inactivity and be as physically active as possible. Fatigue management can be facilitated by nurses and doctors, who can teach patients, their relatives and the public about the recommended interventions [52].

Our study, which included a large sample (*N* = 385) of patients with various diagnoses and applied predetermined thresholds to indicate poor HRQoL of clinical importance [16,17], is a valuable contribution to previous research conducted in Uganda more than 10 years ago [42]. We acknowledge that the study has some limitations. The cross-sectional design did not allow patient follow-up and causation was not established. Despite an adequate study sample, subsamples were relatively small and under-powered. The clinical thresholds were developed in high-income countries with easy access to health care and well-resourced health facilities, in contrast to the setting in our study [17,53]. Furthermore, because we lacked information regarding the dates of patients’ diagnoses, it was not possible to adjust for changes over time. The generalisability of the results to patients who speak languages other than Luganda and English can be questioned [54,55].

## Conclusion

In this study, a high proportion of patients in specialised cancer care in Uganda self-reported HRQoL as poor with impaired physical (79%) and emotional functioning (61%). Four out of five patients reported pain. Improvement of cancer care in East Africa requires a comprehensive and integrated approach that addresses various challenges specific to the region. Such strategies include investment in healthcare infrastructure, e.g. clinical guidelines to improve pain management, and patient education and support services.

## Supplementary Material

Manuscript_changes_highlighted_February_2024.docx

## References

[1] Lin L, Li Z, Yan L, Liu Y, Yang H, Li H. Global, regional, and national cancer incidence and death for 29 cancer groups in 2019 and trends analysis of the global cancer burden, 1990–2019. J Hematol Oncol. 2021;14:1–9. doi: 10.1186/s13045-021-01213-z 34809683 PMC8607714

[2] Sung H, Ferlay J, Siegel RL, Laversanne M, Soerjomataram I, Jemal A, et al. Global cancer statistics 2020: GLOBOCAN estimates of incidence and mortality worldwide for 36 cancers in 185 countries. CA Cancer J Clin. 2021;71:209–49. doi: 10.3322/caac.21660 33538338

[3] Ngwa W, Addai BW, Adewole I, Ainsworth V, Alaro J, Alatise OI, et al. Cancer in sub-Saharan Africa: a lancet oncology commission. Lancet Oncol. 2022;23:e251–e312. doi: 10.1016/S1470-2045(21)00720-8 35550267 PMC9393090

[4] Asasira J, Lee S, Tran TXM, Mpamani C, Wabinga H, Jung S-Y, et al. Infection-related and lifestyle-related cancer burden in Kampala, Uganda: projection of the future cancer incidence up to 2030. BMJ Open. 2022;12:e056722. doi: 10.1136/bmjopen-2021-056722 PMC8928275 35296484

[5] Torre LA, Bray F, Siegel RL, Ferlay J, Lortet‐Tieulent J, Jemal A. Global cancer statistics, 2012. CA Cancer J Clin. 2015;65:87–108. doi: 10.3322/caac.21262 25651787

[6] Ferlay J, Colombet M, Soerjomataram I, Mathers C, Parkin DM, Piñeros M, et al. Estimating the global cancer incidence and mortality in 2018: GLOBOCAN sources and methods. Int J Cancer. 2019 Apr 15;144:1941–53. doi: 10.1002/ijc.31937 30350310

[7] Uganda Cancer Institute [Internet]. Available from: https://uci.or.ug/

[8] World Health Organization. The International Agency for Research on Cancer (IARC). Latest global cancer data: cancer burden rises to 19.3 million new cases and 10.0 million cancer deaths in 2020. Available from: https://www.iarc.who.int/featured-news/latest-global-cancer-data-cancer-burden-rises-to-19-3-million-new-cases-and-10-0-million-cancer-deaths-in-2020/

[9] Ijoma U, Unaogu N, Onyeka T, Nwatu C, Onyekonwu C, Onwuekwe I, et al. Health-related quality of life in people with chronic diseases managed in a low-resource setting–a study from South East Nigeria. Niger J Clin Pract. 2019;22:1180–1188. 31489851 10.4103/njcp.njcp_29_19

[10] Muhamed AN, Chekole B, Tafesse FE, Dessie G, Bantie B, Habtu BF, et al. Quality of life among Ethiopian cancer patients: a systematic review of literatures. Sage Open Nurs. 2023;9:23779608231202691. doi: 10.1177/23779608231202691 37808099 PMC10559689

[11] Okuku F, Orem J, Holoya G, De Boer C, Thompson CL, Cooney MM. Prostate cancer burden at the Uganda cancer institute. J Glob Oncol. 2016;2:181–5. doi: 10.1200/JGO.2015.001040 28717700 PMC5497618

[12] Osann K, Hsieh S, Nelson EL, Monk BJ, Chase D, Cella D, et al. Factors associated with poor quality of life among cervical cancer survivors: implications for clinical care and clinical trials. Gynecol Oncol. 2014;135:266–72. doi: 10.1016/j.ygyno.2014.08.036 25192629 PMC4479396

[13] Fayers PM, Machin D Quality of life: the assessment, analysis and reporting of patient-reported outcomes. 3rd ed. Chichester: Wiley Blackwell; 2016.

[14] Husson O, de Rooij BH, Kieffer J, Oerlemans S, Mols F, Aaronson NK, et al. The EORTC QLQ‐C30 summary score as prognostic factor for survival of patients with cancer in the “real‐world”: results from the population‐based PROFILES registry. Oncology. 2020;25:e722–e32. 10.1634/theoncologist.2019-0348 PMC7160310 32297435

[15] Aaronson NK, Ahmedzai S, Bergman B, Bullinger M, Cull A, Duez NJ, et al. The European organization for research and treatment of cancer QLQ-C30: a quality-of-life instrument for use in international clinical trials in oncology. J Natl Cancer Inst. 1993;85:365–76. doi: 10.1093/jnci/85.5.365 8433390

[16] Giesinger JM, Kuijpers W, Young T, Tomaszewski KA, Friend E, Zabernigg A, et al. Thresholds for clinical importance for four key domains of the EORTC QLQ-C30: physical functioning, emotional functioning, fatigue and pain. Health Qual Life Outcomes. 2016;14:1–8. doi: 10.1186/s12955-016-0489-4 27267486 PMC4897949

[17] Giesinger JM, Loth FL, Aaronson NK, Arraras JI, Caocci G, Efficace F, et al. Thresholds for clinical importance were established to improve interpretation of the EORTC QLQ-C30 in clinical practice and research. J Clin Epidemiol. 2020;118:1–8. doi: 10.1016/j.jclinepi.2019.10.003 31639445

[18] Kaplan RM, Hays RD. Health-related quality of life measurement in public health. Annu Rev Public Health. 2022;43:355–373. doi: 10.1146/annurev-publhealth-052120-012811 34882431

[19] Moradi N, Poder TG, Safari H, Mojahedian MM, Ameri H. Psychometric properties of the EQ-5D-5L compared with EQ-5D-3L in cancer patients in Iran. Front Oncol. 2022;12:1052155. doi: 10.3389/fonc.2022.1052155 36568223 PMC9782428

[20] Araya LT, Fenta TG, Sander B, Gebremariam GT, Gebretekle GB. Health-related quality of life and associated factors among cervical cancer patients at Tikur Anbessa specialized hospital, Addis Ababa, Ethiopia. Health Qual Life Outcomes. 2020;18:1–9. doi: 10.1186/s12955-020-01319-x 32178681 PMC7076924

[21] Katumba J, Obore S, Kaye DK. Health‐related quality of life among patients with ovarian cancer at Mulago Hospital, Uganda. Intl J Gynecol Obstet. 2013;122:115–117. doi: 10.1016/j.ijgo.2013.03.017 23759274

[22] Masika GM, Wettergren L, Kohi TW, von Essen L. Health-related quality of life and needs of care and support of adult Tanzanians with cancer: a mixed-methods study. Health Qual Life Outcomes. 2012;10:133. doi: 10.1186/1477-7525-10-133 23121718 PMC3541157

[23] Mvunta DH, August F, Dharsee N, Mvunta MH, Wangwe P, Ngarina M, et al. Quality of life among cervical cancer patients following completion of chemoradiotherapy at ocean road cancer institute (ORCI) in Tanzania. BMC Womens Health. 2022;22:426. doi: 10.1186/s12905-022-02003-6 36303143 PMC9615158

[24] Shajahan Ahamed M, Degu A. Health-related quality of life among cervical cancer patients at Kenyatta National Hospital. J Oncol Pharm Pract. 2023;29:393–400. doi: 10.1177/10781552211073886 35018848

[25] Sibhat SG, Fenta TG, Sander B, Gebretekle GB. Health-related quality of life and its predictors among patients with breast cancer at Tikur Anbessa Specialized Hospital, Addis Ababa, Ethiopia. Health Qual Life Outcomes. 2019;17:1–10. doi: 10.1186/s12955-019-1239-1 31690327 PMC6833153

[26] Selman LE, Higginson IJ, Agupio G, Dinat N, Downing J, Gwyther L, et al. Quality of life among patients receiving palliative care in South Africa and Uganda: a multi-centred study. Health Qual Life Outcomes. 2011;9:1–14. doi: 10.1186/1477-7525-9-21 21477274 PMC3094195

[27] Wondie Y, Hinz A. Quality of life among Ethiopian cancer patients. Support Care Cancer. 2020;28:5469–78. doi: 10.1007/s00520-020-05398-w 32170441 PMC7547027

[28] Tadele N. Evaluation of quality of life of adult cancer patients attending tikur anbessa specialized referral hospital, Addis Ababa Ethiopia. Ethiop J Health Sci. 2015;25:53–62. doi: 10.4314/ejhs.v25i1.8 25733785 PMC4337080

[29] Ayana BA, Negash S, Yusuf L, Tigeneh W, Haile D. Health related quality of life of gynaecologic cancer patients attending at Tikur Anbesa Specialized Hospital (TASH), Addis Ababa, Ethiopia. BMC Women’s Health. 2018;18:1–9. doi: 10.1186/s12905-017-0507-7 29304790 PMC5755021

[30] Nakaganda A, Solt K, Kwagonza L, Driscoll D, Kampi R, Orem J. Challenges faced by cancer patients in Uganda: implications for health systems strengthening in resource limited settings. J Cancer Policy. 2021;27:100263. doi: 10.1016/j.jcpo.2020.100263 35559936

[31] Koller M, Aaronson NK, Blazeby J, Bottomley A, Dewolf L, Fayers P, et al. Translation procedures for standardised quality of life questionnaires: the European organisation for research and treatment of cancer (EORTC) approach. Eur J Cancer. 2007;43:1810–20. doi: 10.1016/j.ejca.2007.05.029 17689070

[32] Wettergren L, Eriksson LE, Abrahamsson Tornefors Y Translation report of the EORTC QLQ-C30 into Luganda language. City, University of London: 2015. 2015. https://openaccess.city.ac.uk/id/eprint/13247/

[33] Naamala A, Eriksson LE, Orem J, Nalwadda GK, Kabir ZN, Wettergren L. Psychometric properties of the EORTC QLQ-C30 in Uganda. Health Qual Life Outcomes. 2021;19:131. doi: 10.1186/s12955-021-01769-x 33892718 PMC8066473

[34] StataCorp. Stata statistical software: release 18. College Station, TX: StataCorp LCC; 2023.

[35] Yousefi M, Nahvijou A, Sari AA, Ameri H. Mapping QLQ-C30 onto EQ-5D-5L and SF-6D-V2 in patients with colorectal and breast cancer from a developing country. Value Health Reg Issues. 2021;24:57–66. doi: 10.1016/j.vhri.2020.06.006 33508752

[36] Poverty trends: Global, regional and national. UK: fact sheet, 2019. Bristol: Development Initiatives Poverty Research Ltd; 2019. Available from: https://devinit-prod-static.ams3.cdn.digitaloceanspaces.com/media/documents/Poverty_trends_-_global_regional_and_national.pdf

[37] Poverty trends: global, regional and national. Factsheet. Bristol: Development Initiatives Poverty Research Ltd; 2021. Available from: https://devinit-prod-static.ams3.cdn.digitaloceanspaces.com/media/documents/Poverty_trends_-_global_regional_and_national_-_November_2021.pdf.

[38] Christensen Z. Economic poverty trends: global, regional and national. Bristol: Development Initiatives Poverty Research Ltd; 2023.

[39] Owori M. Poverty in Uganda: national and regional data and trends. Development Initiatives. 2020. Available from: https://devinit.org/resources/poverty-uganda-national-and-regional-data-and-trends/

[40] Boyle P, Ngoma T, Sullivan R, Brawley O. Cancer in Africa: the way forward. Ecancermedicalscience. 2019;13:953. doi: 10.3332/ecancer.2019.953 31552126 PMC6722114

[41] Haileselassie W, Mulugeta T, Tigeneh W, Kaba M, Labisso WL. The situation of cancer treatment in Ethiopia: challenges and opportunities. J Cancer Prev. 2019;24:33. doi: 10.15430/JCP.2019.24.1.33 30993093 PMC6453587

[42] Li Z, Aninditha T, Griene B, Francis J, Renato P, Serrie A, et al. Burden of cancer pain in developing countries: a narrative literature review. Clinicoecon Outcomes Res. 2018;10:675. doi: 10.2147/CEOR.S181192 30464561 PMC6219110

[43] Swarm RA, Paice JA, Anghelescu DL, Are M, Bruce JY, Buga S, et al. Adult cancer pain, version 3.2019, NCCN clinical practice guidelines in oncology. J Natl Compr Cancer Netw. 2019;17:977–1007. doi: 10.6004/jnccn.2019.0038 31390582

[44] Derogar M, van der Schaaf M, Lagergren P. Reference values for the EORTC QLQ-C30 quality of life questionnaire in a random sample of the Swedish population. Acta Oncol. 2012;51:10–6. doi: 10.3109/0284186X.2011.614636 21961499

[45] Mols F, Husson O, Oudejans M, Vlooswijk C, Horevoorts N, van de Poll-Franse LV. Reference data of the EORTC QLQ-C30 questionnaire: five consecutive annual assessments of approximately 2000 representative Dutch men and women. Acta Oncol. 2018;57:1381–91. doi: 10.1080/0284186X.2018.1481293 29912607

[46] Waldmann A, Schubert D, Katalinic A, Janda M. Normative data of the EORTC QLQ-C30 for the German population: a population-based survey. PloS One. 2013;8:e74149. doi: 10.1371/journal.pone.0074149 24058523 PMC3769241

[47] Enien MA, Ibrahim N, Makar W, Darwish D, Gaber M. Health-related quality of life: impact of surgery and treatment modality in breast cancer. J Cancer Res Ther. 2018;14:957–63. doi: 10.4103/0973-1482.183214 30197331

[48] Kingham TP, Alatise OI, Vanderpuye V, Casper C, Abantanga FA, Kamara TB, et al. Treatment of cancer in sub-saharan Africa. Lancet Oncol. 2013;14:e158–e67. 23561747 10.1016/S1470-2045(12)70472-2

[49] Germans N, Ellis P, Wilson S, Merriman A, Rabwoni M. The socioeconomic burden of a diagnosis of cervical cancer in women in rural Uganda: findings from a descriptive qualitative study. Intl J Palliat Nurs. 2022;28:322–332. doi: 10.12968/ijpn.2022.28.7.322 35861442

[50] Shen MJ, Prigerson HG, Ratshikana-Moloko M, Mmoledi K, Ruff P, Jacobson JS, et al. Illness understanding and end-of-life care communication and preferences for patients with advanced cancer in South Africa. J Global Oncol. 2018;4:1–9. 10.1200/JGO.17.00160 PMC6223439 30241251

[51] Thong MS, van Noorden CJ, Steindorf K, Arndt V. Cancer-related fatigue: causes and current treatment options. Curr Treat Options Oncol. 2020;21:1–19. doi: 10.1007/s11864-020-0707-5 32025928 PMC8660748

[52] Campbell KL, Winters-Stone K, Wiskemann J, May AM, Schwartz AL, Courneya KS, et al. Exercise guidelines for cancer survivors: consensus statement from international multidisciplinary roundtable. Med Sci Sports Exerc. 2019;51:2375. doi: 10.1249/MSS.0000000000002116 31626055 PMC8576825

[53] Ramasubbu SK, Pasricha RK, Nath UK, Rawat VS, Das B. Quality of life and factors affecting it in adult cancer patients undergoing cancer chemotherapy in a tertiary care hospital. Cancer Rep. 2021;4:e1312. doi: 10.1002/cnr2.1312 PMC8451381 33295136

[54] Namyalo S, Nakayiza J. Dilemmas in implementing language rights in multilingual Uganda. Curr Issues Lang Plan. 2015;16:409–424. doi: 10.1080/14664208.2014.987425

[55] Naamala A, Eriksson LE, Orem J, Nalwadda N, Kabir Z, Wettergren L Health-related quality of life among adult patients with cancer in Uganda. Research Square [Preprint]. 2022 Jun. 10.21203/rs.3.rs-1750279/v1 PMC11008308 38596846

